# The unspecific control of cardiac output during exercise and in (patho‐)physiology: Time to get more specific!

**DOI:** 10.1113/EP092578

**Published:** 2025-03-25

**Authors:** Eric J. Stöhr

**Affiliations:** ^1^ COR‐HELIX (CardiOvascular Regulation and Human Exercise Laboratory – Integration and Xploration), institute of Sport Science Leibniz University Hannover Hannover Germany

You can't reason with your heart; it has its own laws and thumps about things which the intellect scorns.

*A Connecticut Yankee in King Arthur's Court*, Mark Twain (1889)

Ever since William Harvey's conclusion in 1628 that the volume of blood passing through the arteries could not be produced continuously by the liver but that, instead, blood had to be ‘circulating’, cardiac output has become one of the fundamental variables in physiology (Carty, 2016). It has been implicated in the matching of an increased O_2_ demand during exercise, plays an important role in the prevailing blood pressure, and is a major factor when considering cardiovascular disease, for example during heart failure (Bada et al., 2012; Edward et al., 2023; Magder, 2018). Since cardiac output is the product of heart rate and stroke volume, it could be expected that the two factors will be controlled interdependently to arrive at a specific cardiac output (Stöhr, 2022). Support for this thinking has been provided by a study that has shown that atrial pacing above an individual's heart rate did not increase cardiac output during exercise (Munch et al., 2014). Furthermore, peripheral vasodilatation via ATP infusion or heat stress has been said to determine a specific cardiac output, suggesting that cardiac output is a responder to peripheral flow (Bada et al., 2012; Watanabe et al., 2024). However, some gaps in our knowledge remain. For example, increasing the heart's chronotropy via atrial pacing without concomitant sympathetic stimulation will remove the typical influence on myocardial contractility and vasomotor tone. Equally, peripheral vasodilatation via ATP is insightful but does not represent the in vivo effects of peripheral vasodilatation caused by simultaneous neurohormonal control. Therefore, previous findings may not be transferrable to the natural in vivo regulation. And while the relationship between cardiac output and peripheral flow as a result of heat stress is undisputed, it remains unclear whether cardiac output determines the peripheral flow or vice versa. Far from being a trivial matter, a few scenarios are thinkable: an increased peripheral flow could be caused by (1) rate‐induced changes in the frequency of cardiac ejection, (2) volume‐induced changes by enhanced stroke volume of the heart, or (3) volume‐induced regulation by peripheral vasodilatation. While the latter scenario is most likely the initiator via rapid, local dilatation of the microcirculation of the skin or the skeletal muscle capillaries involving gap junctions composed of Cx40 (Kowalewska et al., 2024), most recent studies have not been able to detect a significant change in the proximal conduit artery diameter (Watanabe et al., 2024). Until the beat‐by‐beat time course of events along the O_2_ cascade is shown, empirical evidence for scenario (3) remains theoretical. Coupled with the aforementioned doubt that cardiac output is indeed specific and ‘matches’ the peripheral demand (and flow) via coordinated control of stroke volume and heart rate, the control of cardiac output in different conditions deserves ongoing investigation. For this reason, *Experimental Physiology* has put together a collection of 12 articles in a special issue entitled ‘The unspecific control of cardiac output during exercise and in (patho‐)physiology*’*.

The different perspectives in the special issue represent the contemporary complexity of cardiac physiology. Whether the healthy heart can indeed be seen as a ‘secondary organ’ within an open‐loop circulatory system is an important matter of debate (Furst & González‐Alonso, 2025; Joyner, 2025). (It is often thought that the circulatory system is closed. However, this is not strictly the case, because plasma volume is constantly altered by hydration and there is permanent intra‐extracellular exchange of H_2_O.) The implications of a potential mis‐control of cardiac output in heart failure is another important area (Cornwell, 2025; O'Leary & Mannozzi, 2025; Sagmeister et al., 2025). Equally, the impact of a healthy chronic adaptation to exercise training and the interaction between cardiac output and cerebral blood flow during exercise is of great interest (Fischer, Jeppesen et al., 2025; Ogoh, 2025). Dr Heinonen discusses whether the maximal cardiac output during exercise may be dependent on myocardial blood flow and proposes the intriguing hypothesis that the maximal heart rate of athletes may be limited to protect the heart from ischaemia (Heinonen, 2025). This highlights how important it is to understand the interplay between heart rate and stroke volume in different conditions and that we still do not fully appreciate the extent of their interdependency in relation to the overall output of the heart. Further discussions in the special issue include the effects of altitude and ischaemia, specifically the potential for mitochondrial transplantation in ischaemia–reperfusion injury (Bafadam et al., 2025; Zhu et al., 2025). In the final two articles, the role of sympathetic restraint and the specificity of cardiac output during heat stress and exercise are discussed. Drouin et al. (2025) highlight that the concept of sympathetic restraint during exercise needs to consider two aspects that have previously received too little attention. The consequences of their deliberations add important insight into the debate whether the cardiovascular system may be ‘cardiocentric’ or ‘peripherocentric’ (Drouin et al., 2025), as also highlighted by other authors (Furst & González‐Alonso, 2025; Joyner, 2025). Lampkemeyer et al. (2025) provide new evidence for a limited matching of cardiac output during thermal stress and the metabolic demands of exercise. The findings contradict the longstanding expectation that the cardiac output of healthy humans is specific to the whole‐body demands of fundamental biological conditions. As a direct consequence, the ‘inviolate relationship’ of ‘5–6 L/min of cardiac output … required for every litre of oxygen uptake above rest’ (Thompson, 2001) needs to be revisited. It is clear that such an estimate is not precise enough for our contemporary understanding (Figure 1).

**FIGURE 1:**
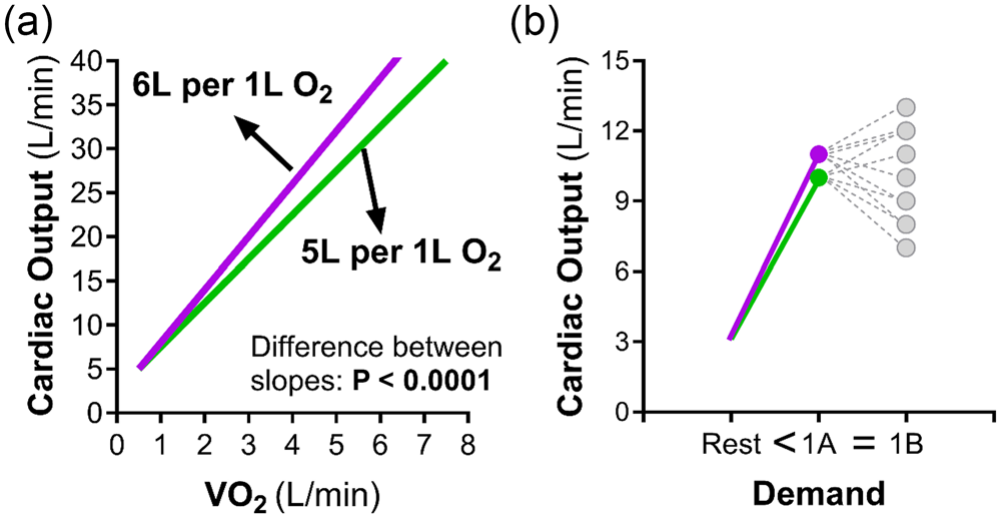
The unspecific cardiac output. (a) The long‐standing assumption that cardiac output is 5–6 L per L O_2_ is not precise enough, since the two relationships are significantly different from one another. (b) When a new cardiac output above resting levels has been reached for a specific condition (1A), this cardiac output should be maintained if the demand did not change. However, most often, small deviations in either heart rate or stroke volume are not precisely ‘compensated’ for by the other factor and hence a different cardiac output is generated (grey circles), despite the same demand (1B).

So where does this leave us in terms of improving our understanding of the physiology of the heart?

Collectively, the evidence presented in this special issue suggests that the control of heart rate and stroke volume occurs less interdependently as previously proposed and that the resultant cardiac output may be unspecific due to factors that have eluded us so far. In this context, a picture emerges that suggests that the volumetric component of cardiac output may be more dependent on arterial and venous haemodynamics than previously thought. Such insight must not be confounded with the established ideas proposed by landmark concepts such as that by Guyton (1955). Rather, considering more recent evidence that shows that contraction and relaxation of the heart muscle may not relate as closely to stroke volume and filling as often assumed, future work should gain an improved understanding of the complex systolic and diastolic cardiac and arterial performances (Cooke et al., 2018; Fischer, Bonne et al., 2025; Samuel & Stöhr, 2017). The respective cardiac contraction and relaxation dynamics may also influence some of the pulmonary, aortic and peripheral haemodynamics, for example through suction forces and variable ejection dynamics. It seems that to really understand the heart's ‘own laws’ and to discover novel principles beyond ‘things which the intellect scorns’ (Twain, 1889), future studies should include measurements of individuals’ cardiac contractile dynamics, different intra‐ventricular pressure developments, intra‐aortic pressure patterns, specific venous haemodynamics and the interplay between heart rate and stroke volume. In this regard, the contemporary evidence presented in this special issue will hopefully motivate researchers to advance our knowledge and get more specific – and find out *why* the heart really produces a certain output with every carefully coordinated contraction.

## AUTHOR CONTRIBUTIONS

Sole author.

## CONFLICT OF INTEREST

The author acknowledges that he has co‐authored two articles that have appeared in the special issue. These articles were reviewed independently, by external reviewers.

## FUNDING INFORMATION

No funding was received for this work.
